# Prevalence and factors associated with sarcopenia in elderly people referred by Primary Healthcare to a specialized service in an area of the Federal District of Brazil, 2015-2017

**DOI:** 10.1590/S2237-96222025v34e20240500.en

**Published:** 2025-08-04

**Authors:** Hudson Azevedo Pinheiro, Vera Regina Cerceau, Luana de Azevedo Pinheiro, Valéria Pagotto, Ruth Losada de Menezes

**Affiliations:** 1 Secretaria de Saúde do Distrito Federal, Diretoria Regional de Atenção Secundária, Brasília, DF, Brazil; 2 Universidade Católica de Brasília, Curso de Enfermagem, Brasília, DF, Brazil; 3 Universidade Federal de Goiás, Curso de Enfermagem, Goiânia, GO, Brazil; 4 Universidade Federal de Goiás, Curso de Fisioterapia, Goiânia, GO, Brazil

**Keywords:** Elderly Person, Sarcopenia, Primary Healthcare, Prevalence, Association Measures, Exposure, Risk or Outcome, Anciano, Sarcopenia, Atención Primaria de Salud, Predominio, Medidas de Asociación, Exposición, Riesgo o Resultado

## Abstract

**Objectives:**

To estimate the prevalence of sarcopenia and identify associated factors by sex in elderly people referred by Primary Healthcare to a geriatrics and gerontology reference service in an area of the Federal District in the period of 2015-2017.

**Methods:**

This is a longitudinal study that used the criteria of the European Working Group of Sarcopenia in Older People (hand grip, calf circumference and usual gait speed), in addition to clinical aspects and socioeconomic data.

**Results:**

500 elderly people were evaluated in an area of the Federal District, of which 32.6% were pre-sarcopenic, 32.2% were sarcopenic and 7.6% were severely sarcopenic. Risk factors (>70 years and urinary incontinence) and protection factors (high body mass index) were identified. Heart disease and osteoporosis were also identified as risk factors for women and, for men, visual impairment, and occurrence of falls in the last six months were the risk factors identified before the anamnesis.

**Conclusion:**

The prevalence of 32.2% of sarcopenia and 7.6% of severe sarcopenia was observed in elderly people treated by Primary Healthcare in the Federal District, and age >70 years old and urinary incontinence were identified as risk factors. High body mass index was considered a protective factor. These data highlighted the need for public health strategies aimed at preventing and treating sarcopenia, considering regional particularities and risk factors specific to each community.

## Resumen

**Objetivos:**

Estimar la prevalencia de sarcopenia e identificar factores asociados según sexo en adultos mayores referidos desde Atención Primaria a un servicio de referencia en geriatría y gerontología en una región específica del Distrito Federal en el período 2015-2017.

**Métodos:**

Se realizó un estudio longitudinal en el que se utilizaron los criterios del European Working Group of Sarcopenia in Older People (prensión manual, circunferencia de pantorrilla y velocidad de marcha), además de aspectos clínicos y datos socioeconómicos.

**Resultados:**

Se evaluaron 500 adultos mayores en una región del Distrito Federal, de los cuales 32.6% eran presarcopénicos, 32.2% sarcopénicos y 7.6% sarcopénicos graves. Se identificaron factores de riesgo (>70 años e incontinencia urinaria) y factores protectores (índice de masa corporal elevado). También se identificaron como factores de riesgo las enfermedades cardíacas y la osteoporosis para las mujeres y, para los hombres, la discapacidad visual y la aparición de caídas en los últimos seis meses previos a la anamnesis.

**Conclusión:**

La prevalencia de sarcopenia fue de 32,2% y de sarcopenia grave de 7,6% en los ancianos atendidos en Atención Primaria en el Distrito Federal, siendo identificados como factores de riesgo la edad >70 años y la incontinencia urinaria. Se consideró que un índice de masa corporal elevado era un factor protector. Estos datos destacaron la necesidad de estrategias de salud pública dirigidas a prevenir y tratar la sarcopenia, considerando las particularidades regionales y los factores de riesgo específicos de cada comunidad.

**Ethical aspects**

This research respected ethical principles, having obtained the following approval data::

Research Ethics Committee: Fundação de Ensino e Pesquisa em Ciências da Saúde

Opinion number: 1.128.355

Approval date: 29/6/2015

Certificate of Submission for Ethical Appraisal: 26147019.3.0000.5553

Informed Consent Form: Obtained from all participants prior to collection.

## Introduction

For some time now, there has been frequent discussion about the conceptual model between Primary Healthcare and basic healthcare. Primary Health Care refers to the main gateway that guarantees timely attention to services belonging to the B Brazilian Unified Health System. Basic Healthcare was designed and structured with a focus on promotion, prevention and care aimed at collective health. Misunderstandings are prevalent regarding their meanings, and in practice there is a limbo between access to specialized services and prevention, which is characterized as challenging – especially for elderly people in the current scenario of population growth in this age group (1,2).

The term sarcopenia has been used in routine services for aging people and its clinical diagnosis has always been controversial. In 2010, the European Working Group of Sarcopenia in Older People defined sarcopenia as a muscle disease, which can be acute or chronic, and recommended a new algorithm based on the identification of suspected cases, evaluation, confirmation, and classification of this syndrome by healthcare professionals. This could be implemented in Primary Healthcare, since this condition can lead to loss of independence in activities of daily living, increase the risk of falls, and lead to hospitalization and death in elderly people (3,4).

Sarcopenia affects between 14.0% and 30.0% of individuals aged 60 or over, mainly in populations with greater social and economic vulnerability. This condition is related to lifestyle. Healthy eating habits and regular physical exercise contribute to better control of chronic diseases, such as high blood pressure, diabetes, and obesity, which are within the scope of actions under the responsibility of Primary Healthcare (5-8).

It is important to identify the presence of the sarcopenia phenotype in elderly people living in the community and the factors associated with its emergence to plan strategies and interventions appropriate to the reality of that community. This started with Primary Healthcare itself, aiming to minimize vulnerabilities and comorbidities that could lead these people to a worse quality of life and increase costs for health systems (9,10).

The objective of this study was to estimate the prevalence of sarcopenia, in addition to identifying the factors associated by biological gender in elderly people referred by Primary Healthcare to a reference service in geriatrics and gerontology in a specific area of the Federal District.

## Methods

### Study design

This was a cross-sectional study conducted between September 2015 and December 2017. Considering that the estimated frequency in population studies for sarcopenia is 33.0% of the elderly population, a sample size calculation was performed using the EpiInfo software, version 7. With a significance level of 95.0%, the target to be achieved was 384 subjects.

### Context

In the latest demographic censuses, Brasília emerged as the third largest Brazilian metropolis. The challenges of population aging, especially among the elderly population and in more peripheral areas, motivated this study.

### Participants

We divided Brasília into seven major areas regarding health policies. This study was conducted with the Southwest Regional Health Superintendence of the Federal District (Brazil), at the time comprising the administrative regions and their basic health units: Águas Claras (1), Samambaia (11), Recanto da Emas (7), Taguatinga (8) and Vicente Pires (1). The estimated population was 828,703 inhabitants; 20% were over 60 years old. The per capita income was R$ 1,639.04.

Elderly people who presented issues related to clinical vulnerability when filling out the Seniors Health Booklet (11), were referred to the reference outpatient clinic for geriatrics and gerontology located in the Administrative Region of Taguatinga, through the consultation regulation system (SISreg).

At the outpatient clinic, an interdisciplinary consultation was conducted with a geriatrician, nurse, physiotherapist, and nutritionist who were duly trained for this purpose. The professionals took notes on the medications in use, socioeconomic aspects, previously diagnosed clinical comorbidities, in addition to specific complaints related to vulnerability described in the Seniors Health Booklet of the Ministry of Health, such as the number of falls in the last year, the practice of regular physical activity (at least 150 minutes of weekly practice of any modality was required to count as regular physical activity), the body mass index (BMI), the existence of urinary incontinence, dependence for activities of daily living and the identification of the sarcopenia phenotype.

The consultations were conducted with the participation of family members and caregivers of the elderly patients. Initially, an interview was conducted for health profiling with questions related to income, marital status, and education. There was also ratification of information contained in the reason for referral, such as the presence of urinary incontinence, one or more falls in the last six months before the anamnesis and number of medications in use.

### Inclusion and exclusion criteria

As an inclusion criterion, we considered elderly people who were in vulnerable conditions according to the criteria from the Seniors Health Booklet of the Ministry of Health, identified by the family doctor and family health strategy team during matrix support actions (11). The exclusion criteria adopted were elderly people who presented sequelae of neurological diseases (cerebrovascular disease, parkinsonism, dementia, among others), in addition to subjects with amputations, since such conditions are related to sarcopenia secondary to disuse attributed to such conditions.

### Variables

For the diagnosis of sarcopenia, the recommendation proposed by the European consensus was used through an algorithm that considers three distinct criteria: muscle strength, the quantity or quality of muscle mass and physical performance (3).

The handgrip strength test, performed using a Jamar hydraulic hand dynamometer, was used as a marker of overall muscle strength in elderly people. For the test, the patient sits on a chair without resting his back on it, while keeping his elbow flexed. In the dominant hand, three measurements were taken with a one-minute interval between them. Sarcopenia was considered if the measurements had values lower than 27 kg/F for men and 16 kg/F for women (12,13).

To assess the quality of muscle mass, the circumference of the calf was measured with the patient sitting in a chair, legs relaxed and feet resting on the floor, with knees flexed at 90º. After identifying the most protruding region of the legs, using an inelastic measuring tape, the perimeter was measured, with individuals who presented values equal to or less than 33 cm for women and 34 cm for men being considered at risk (14).

Physical performance, measured by the usual gait speed test, was conducted in a corridor, where the elderly person was instructed to walk at their usual speed, and could use an aid to assist with ambulation. The time taken to move three meters was measured, disregarding acceleration and deceleration times, indicating a risk at speeds lower than 0.8 m/s (15). 

The criteria recommended by the European consensus were used to classify the patients as sarcopenic. In this case, subjects who presented reduced muscle strength and quality of muscle mass were classified as sarcopenic. The classification as severely sarcopenic also required the presence of reduced physical performance (3).

Anthropometric variables were measured in height (cm) and weight (kg) using a Filizola scale with a stadiometer, to then calculate the BMI. Lipschitz’s recommendation was used, with elderly people with values lower than 22 kg/cm^2^ classified as underweight and with values between 22 and 27 kg/cm^2^ classified as eutrophic. Elderly people with a BMI greater than 27 kg/cm^2^ were classified as overweight, and these measures are more sensitive for public health (16).

Then, the Barthel index was applied to identify the degree of functional independence, using the validated and cross-culturally adapted version to the Brazilian population, whose cut-off point was greater than or equal to 60 to determine the subjects as independent for activities of daily living. To identify the presence of symptoms related to urinary incontinence, the urinary rhythm of the Barthel index was used, which considers an elderly person who reaches 10 points to be continent and those who reach 5 or 0 points to be incontinent (17). 

We applied a questionnaire with questions related to socioeconomic aspects (interview for health profiling with questions about income, marital status, education), presence of urinary incontinence, one or more falls in the last six months. before the anamnesis, number of medications in use and level of physical activity.

### Statistical methods

For statistical analysis, the following was used: Stata software version 15. In the descriptive analysis, the variables were weighted according to mean, standard deviation, and proportions. The prevalence ratio for sarcopenia and the 95.0% confidence interval were estimated. 

Sarcopenia was used as an independent variable and, through it, the Poisson test was used to establish the associated factors through the prevalence ratio and the 95.0% confidence interval.

## Results

**Figure 1:**
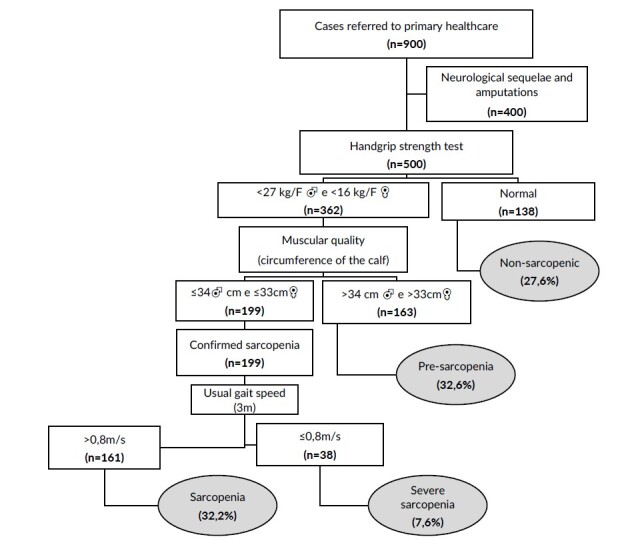
Diagnosis and prevalence of sarcopenia according to the algorithm of the European Working Group on Sarcopenia in Older People

During the collection period, 900 elderly people referred by basic health units to the polyclinic were invited to participate. Of these, 240 were excluded due to having sequelae of a stroke, 50 had a confirmed diagnosis of Parkinson’s disease, 80 had severe cognitive impairment (dementia syndrome) and 30 had suffered limb amputation. Five hundred individuals were evaluated and classified according to the sarcopenia phenotype (Figure 1).

There was prevalence and factors associated with sarcopenia in men and women referred by Primary Healthcare, from the perspective of socioeconomic and behavioral issues, which highlighted risk factors such as age group >70 years old, and protective factors, such as having a partner and having eutrophic BMI or being overweight (Table 1). The practice of physical exercise was confirmed as a protective factor for sarcopenia for women.

**Table 1:** Raw and adjusted prevalence ratio (PR) and 95% confidence interval (95%CI) of sarcopenia in adults referred by Primary Healthcare from the perspective of socioeconomic and behavioral issues. Brasilia, 2017 (n=500)

	Females (n=128)		Males (n=33)	
	RP (95%CI)	p-value	RP (95%CI)	p-value
Socioeconomic aspects				
**Age group** (years)				
60-69	1.00		1.00	
70-79	1.77 (1.29; 2.41)	<0.001	3.37 (1.13; 10.17)	<0.051
>80	2.55 (1.91; 3.40)	<0.001	9.23 (3.35; 25.38)	<0.001
**Years of education**				
No formal education	1.00		1.00	
≤8	0.83 (0.63; 1.08)	0.18	0.93 (0.32; 2.69)	0.891
>8	0.95 (0.69; 1.29)	0.75	0.25 (0.60; 1.08)	0.060
**Living with a partner**				
Yes	0.77 (0.60; 0.99)	<0.05	0.39 (0.19; 0.82)	<0.050
No	1.00		1.00	
**Family income**#				
1-2 minimum wages	1.00		1.00	0.332
>3 minimum wages	1.01 (0.79; 1.31)	0.87	0.67 (0.30; 1.48)	
Behavioral variables				
**Physical activity**				
Yes	0.45 (0.31; 0.66)	<0.001	1.57 (0.91; 2.72)	0.100
**Body Mass Index** (BMI) (kg/cm^2^)				
Low weight	1.00		1.00	
Eutrophic	0.51 (0.40; 0.64)	<0.001	0.46 (0.29; 0.73)	<0.001
Overweight	0.25 (0.19; 0.34)	<0.001	0.18 (0.07; 0.43)	<0.001

Urinary incontinence was a risk factor for sarcopenia in both sexes in relation to clinical aspects (Table 2). For women, the main risk factors for developing sarcopenia were taking more than five medications and having heart disease and osteoporosis. Interestingly, those who had joint problems or hypothyroidism did not have sarcopenia. For men, the factors that identified the presence of the phenotype were visual problems and occurrence of falls in the last six months before the anamnesis.

**Table 2:** Raw and adjusted prevalence ratio (PR) and 95% confidence interval (95%CI) of sarcopenia in adults referred by Primary Healthcare regarding clinical aspects related to sarcopenia. Brasília, 2017 (n=161)

	Females (n=128)		Males (n=33)	
	RP (95%CI)	p-value	RP (95%CI)	p-value
**Medications in use**				
≤4 medications	1.00			
>5 medications	1.32 (1.07; 1.62)	<0.001	1.12 (0.72; 1.73)	0.611
Comorbidities				
High blood pressure	1.26 (0.97; 1.65)	0.077	0.78 (0.48; 1.25)	0.311
Diabetes	1.08 (0.87; 1.34)	0.4799	0.82 (0.51; 1.33)	0.435
Heart disease	1.27 (1.08; 1.61)	<0.050	0.73 (0.42; 1.28)	0.289
Osteoporosis	1.42 (1.16; 1.75)	<0.001	1.10 (0.53; 2.29)	0.789
Hypothyroidism	0.45 (0.31; 0.66)	<0.001	1.57 (0.91; 2.72)	0.100
Depression	0.94 (0.76; 1.17)	0.621	1.35 (0.84; 2.16)	0.201
Joint problems	0.67 (0.51; 0.87)	<0.050	1.12 (0.67; 1.88)	0.641
Cancer	1.54 (0.84; 2.85)	0.151	0.98 (0.49; 1.97)	0.966
Visual problems	1.96 (0.97; 3.96)	0.062	2.65 (1.32; 5.29)	<0.050
Respiratory problems	1.21 (0.78; 1.88)	0.389	0.26 (0.03; 1.90)	0.188
Fall in the last 6 months	1.12 (0.91; 1.38)	0.275	3.31 (1.98; 5.53)	<0.001
Urinary incontinence	1.29 (1.04; 1.59)	<0.050	2.21 (1.42; 3.42)	<0.001

## Discussion

The study observed that, in a specific area of the Federal District, 163 elderly people (32.6%) were pre-sarcopenic, 161 (32.2%) were sarcopenic and 38 (7.6%) were severely sarcopenic. Risk factors were identified, such as >70 years old and urinary incontinence, as well as protective factors, such as high BMI. Heart disease and osteoporosis were indicated for women. For men, visual impairment and the occurrence of falls in the last six months were identified as risk factors.

A European study used a projection model based on the current prevalence of sarcopenia and demographic data available for the population of 28 countries of the European Union. It has been suggested that the number of patients with sarcopenia will increase significantly in the next 30 years, which will have significant consequences, becoming a public health problem (18).

In western China, the prevalence of sarcopenia was compared between urban and rural areas. Among 612 individuals evaluated as a whole, 9.8% presented sarcopenia. When the separate analysis was conducted, the rural area had a prevalence of 13.1%, almost the double of the population living in urban areas (7.0%). It was highlighted that 61.0% of sarcopenic elders lived in rural areas (19).

The present region in the Federal District is made up of farms, where access to second-level healthcare services (polyclinics) for longitudinal monitoring may be compromised. The identification of the sarcopenia phenotype could be implemented in routines and activities conducted in Primary Healthcare for the elderly population.

In Australia, in a sample of 162 elderly people, it was observed that 25 (16.0%) of them were classified as sarcopenic. Sedentary behavior was identified as the main element associated with the decrease in muscle mass, increasing the risk of developing sarcopenia (20).

In this study, women who exercised did not present sarcopenia, which characterized the access to healthcare as a predominantly female behavior. On-site strategies are needed to encourage men to seek Primary Healthcare earlier, not just when they become elderly, so they can maintain their health and not seek care only when they are ill.

Regarding lifestyle, preventing sedentary behavior leads to a lower risk of developing morbidities in elderly people resulting from sarcopenia, as performing physical exercises for at least 150 minutes per week is essential for the development of muscle mass and muscle strength. It has been observed that a low-protein diet, in addition to smoking and alcoholism, leads to loss of muscle mass (21).

It was also shown that elderly people who had a higher income regularly practiced some physical activity, whereas low-income individuals considered occupational activity as physical activity (22-24). Low-income individuals tended to adopt other less healthy habits due to lack of information, such as smoking and alcoholism, which contributed to the development of comorbidities or chronic diseases regardless of gender. These factors may contribute to increased prevalence rates of sarcopenia in the coming years (22-24).

Although the results of this study demonstrate a higher prevalence of sarcopenia when compared to other populations, it is worth remembering that senior persons often present vulnerabilities, whether in access to specialized services or due to cultural issues regarding the aging process, as if losing strength and incontinence were normal for their age. There is a need for Primary Healthcare, which is closest to this population, to develop strategies to identify the risk of sarcopenia and to monitor the condition through preventive, educational and monitoring actions.

Sarcopenia negatively influences the recovery of independence in urination and defecation, regardless of physical and cognitive level (25,26). Early detection of sarcopenia and its treatment through dietary modifications and reduction of sedentary behavior should be implemented to predict and maximize improvement in independence, since sphincter control is an activity of daily living (25,26).

There is no consensus in the literature about whether a particular biological gender is more predisposed to sarcopenia than another. In men, the decline of sex steroids is much slower than in women, which may be a key factor in explaining the significantly higher prevalence of sarcopenia in women between 60 and 70 years of age. In men, after the eighth decade of life, testosterone concentrations decrease rapidly, which can contribute to the emergence of sarcopenia. Women, on average, have a longer life expectancy than men due to the culture of care and prevention, regularly seeking health services such as Primary Healthcare (27,28).

The greatest risk of becoming sarcopenic is when the elderly person has more than two conditions (multimorbidities), especially diabetes, which can cause an increase in insulin resistance and, therefore, intensify the loss of muscle mass (7). 

There is no consensus on the cause-and-effect relationship between sarcopenia and osteoporosis. For the development of the bone matrix, muscle contraction and the implementation of muscle load exercises are necessary, which are also indicated for the treatment of sarcopenia. Among the strategies related to preventing falls, which are essential for preventing fractures due to osteoporosis, strength exercises are also recommended (29,30). 

A correlation between the risk of falls and sarcopenia was found in 27.0% of men and 20.0% of women (31). One fact that may have indicated a greater risk of falling in men when compared to women was the regular practice of physical exercise, which, in the case of females, is frequent and has proven to be a protective factor for both sarcopenia and joint pain.

Elderly people who have a partner were highlighted in the study as a protective factor for sarcopenia, demonstrating the need for social interaction as a basic human need. Having someone with you can function as a strategy for motivation and facilitate persuasion regarding the prevention of risky behaviors, mainly due to the health and care guidelines that are part of the routine of Primary Healthcare. There are also coexistence practices, such as the Hiperdia hypertension and diabetes prevention program implemented by the SUS (Brazilian Unified Health System), and integrative and complementary health practices that can contribute to the prevention of sarcopenia, since the elderly person needs to go to the basic health unit to participate and coexist; therefore, the risk of social isolation also decreases.

Clinical guidelines for the treatment of sarcopenia are still lacking in Brazil; however, prevention through regular physical exercise and a balanced diet provide quality of life for successful aging. Sarcopenia entails enormous costs for society; in turn, the increase in life expectancy and the consequent increase in the elderly population have caused a significant prevalence of sarcopenia. Cost-effective management of this disease has become particularly important (32). 

Ordinance No. 635/2023 of the Brazilian Ministry of Health established the federal financial incentive for the implementation and funding of multidisciplinary teams in Primary Healthcare. This incentive came at a time of reconstruction focused on basic care for people throughout their lives, through the strengthening of interprofessional actions and the incorporation of technologies and innovations in health to promote comprehensive care for the population. This contributes to expanding the scope of practices and outcomes in Primary Healthcare itself (33).

As limitations of this study, information on lifestyle such as smoking, alcohol consumption and leisure activities was not collected, as well as race/skin color, religion, and nutritional status. This information could determine other factors that may influence the development of sarcopenia. In addition, we could not identify which basic health units were responsible for referring elderly people with sarcopenia, a fact that could help identify the areas of greatest vulnerability for matrix support strategies.

This study observed the prevalence of 32.2% of sarcopenia and 7.6% of severe sarcopenia in senior people treated by Primary Healthcare in a specific area of the Federal District. This has been considered a real public health problem today and risk factors identified were being >70 years old and having urinary incontinence, whereas having a high BMI functioned as a protective factor.

Heart disease and osteoporosis were risk factors for sarcopenia in women and, for men, the risk factors were visual problems and occurrence of falls. As protective factors against sarcopenia for women, having a partner and performing regular physical activity were identified.

## Data Availability

The database may be made available in an Excel spreadsheet upon request to the corresponding author.
