# Supplementary material for: Prevalence and factors associated with sarcopenia in elderly people referred by Primary Healthcare to a specialized service in an area of the Federal District of Brazil, 2015-2017
Source: Epidemiol Serv Saude. 2025 Aug 4;34:e20240500. doi: 10.1590/S2237-96222025v34e20240500.en (PMC12334157; doi:10.1590/S2237-96222025v34e20240500.en)
Supplement: Translation

# Translation: Prevalência e fatores associados à sarcopenia em pessoas idosas encaminhadas pela Atenção Primária em Saúde a um serviço especializado em determinada região do Distrito Federal, 2015-2017

**DOI:** 10.1590/S2237-96222025v34e20240500.pt

**Authors:** Hudson Azevedo Pinheiro, Vera Regina Cerceau, Luana de Azevedo Pinheiro, Valéria Pagotto, Ruth Losada de Menezes

**Affiliations:** 1 Secretaria de Saúde do Distrito Federal, Diretoria Regional de Atenção Secundária, Brasília, DF, Brasil; 2 Universidade Católica de Brasília, Curso de Enfermagem, Brasília, DF, Brasil; 3 Universidade Federal de Goiás, Curso de Enfermagem, Goiânia, GO, Brasil; 4 Universidade Federal de Goiás, Curso de Fisioterapia, Goiânia, GO, Brasil

**Keywords:** Pessoa Idosa, Sarcopenia, Atenção Primária à Saúde, Prevalência, Medidas de Associação, Exposição, Risco ou Desfecho

## Abstract

**Objetivos:**

Estimar a prevalência de sarcopenia e identificar os fatores associados por sexo em pessoas idosas encaminhadas pela Atenção Primária a um serviço de referência em geriatria e gerontologia em determinada região do Distrito Federal no período 2015-2017.

**Métodos:**

Tratou-se de estudo longitudinal que utilizou os critérios do European Working Group of Sarcopenia in Older People (preensão palmar, circunferência de panturrilha e velocidade de marcha), além de aspectos clínicos e dados socioeconômicos.

**Resultados:**

Foram avaliadas 500 pessoas idosas em uma região do Distrito Federal, sendo 32,6% pré-sarcopênicos, 32,2% sarcopênicos e 7,6% sarcopênicos graves. Identificaram-se fatores de risco (>70 anos e incontinência urinária) e de proteção (índice de massa corporal elevado). Também foram apontados como fatores de risco cardiopatias e osteoporose para as mulheres e, para os homens, déficit visual e ocorrência de quedas nos últimos seis meses antes da anamnese.

**Conclusão:**

Observaram-se as prevalências de 32,2% de sarcopenia e 7,6% de sarcopenia grave em idosos atendidos pela Atenção Primária no Distrito Federal, e identificaram-se como fatores de risco a idade >70 anos e incontinência urinária. O índice de massa corporal elevado foi considerado como fator de proteção. Esses dados destacaram a necessidade de estratégias de saúde pública direcionadas para a prevenção e o tratamento da sarcopenia, considerando as particularidades regionais e os fatores de risco específicos de cada comunidade.

**Aspectos éticos**

A presente pesquisa respeitou os princípios éticos, obtendo os seguintes dados de aprovação: :

Comitê de ética em pesquisa: Fundação de Ensino e Pesquisa em Ciências da Saúde

Número do parecer: 1.128.355

Data de aprovação: 29/6/2015

Certificado de apresentação de apreciação ética: 26147019.3.0000.5553

Registro do consentimento livre e esclarecido: Obtido de todos os participantes antes da coleta.

## Introdução

Há algum tempo, existe a discussão frequente sobre o modelo conceitual entre Atenção Primária à Saúde e atenção básica. A Atenção Primária à Saúde se refere à principal porta de entrada que garante atenção oportuna aos serviços pertencentes ao Sistema Único de Saúde. A atenção básica foi idealizada e estruturada com enfoques para a promoção, a prevenção e o cuidado visando à saúde coletiva. Equívocos são predominantes quanto aos significados, e observam-se na prática o limbo entre o acesso aos serviços especializados e a prevenção, que se caracteriza como desafiadora – principalmente às pessoas idosas no atual cenário de aumento populacional nesse ciclo de vida (1,2).

O termo sarcopenia tem sido utilizado nas rotinas de serviços ligados ao envelhecimento e o seu diagnóstico clínico sempre foi controverso. Em 2010, o European Working Group of Sarcopenia in Older People definiu sarcopenia como doença muscular, que pode ser aguda ou crônica, e recomendou novo algoritmo com base na identificação de casos suspeitos, avaliação, confirmação e classificação da presente síndrome por parte dos profissionais de saúde. Isso pôde ser implementado na Atenção Primária, uma vez que essa condição pode levar à perda de independência das atividades de vida diária, aumentar o risco de ocorrerem quedas, levar à hospitalização e morte em pessoas idosas (3,4).

A sarcopenia atinge entre 14,0% e 30,0% dos indivíduos com 60 anos ou mais, principalmente nas populações de maior vulnerabilidade social e econômica. Essa condição está relacionada ao estilo de vida. Hábitos alimentares saudáveis e prática regular de exercícios físicos contribuem para o melhor controle de doenças crônicas, como hipertensão arterial, diabetes e obesidade, que estão no escopo de ações de responsabilidade da Atenção Primária à Saúde (5-8).

É importante identificar a presença do fenótipo sarcopenia em pessoas idosas residentes na comunidade e quais são os fatores associados ao seu surgimento para o planejamento de estratégias e intervenções adequadas à realidade daquela comunidade. Iniciou-se pela própria Atenção Primária, visando minimizar vulnerabilidades e comorbidades que possam levar essas pessoas à pior qualidade de vida e onerar custos aos sistemas de saúde (9,10).

O objetivo deste estudo foi estimar a prevalência de sarcopenia, além de identificar os fatores associados por sexo em pessoas idosas encaminhadas pela Atenção Primária a um serviço de referência em geriatria e gerontologia em determinada região do Distrito Federal.

## Métodos

### Delineamento

Tratou-se de estudo transversal realizado entre setembro de 2015 e dezembro de 2017. Levando em consideração que a frequência estimada em estudos populacionais para a sarcopenia é de 33,0% da população idosa, foi realizado cálculo de tamanho de amostra utilizando o software EpiInfo versão 7. Com nível de significância de 95,0%, a meta a ser atingida era de 384 sujeitos.

### Contexto

Nos últimos censos demográficos, Brasília surgiu como a terceira maior metrópole brasileira. Os desafios sobre o envelhecimento populacional, sobretudo na população idosa e em regiões mais periféricas, motivaram este estudo.

### Participantes

Houve divisão em sete grandes áreas em relação às políticas de saúde em Brasília. Este estudo foi conduzido com a Superintendência Regional de Saúde Sudoeste do Distrito Federal, à época composta pelas regiões administrativas e suas unidades básicas de saúde: Águas Claras (1), Samambaia (11), Recanto da Emas (7), Taguatinga (8) e Vicente Pires (1). A população estimada foi 828.703 habitantes; 20% tinham mais de 60 anos. A renda per capita era R$ 1.639,04.

As pessoas idosas que apresentassem quesitos relacionados à vulnerabilidade clínica, por meio do preenchimento da Caderneta de Saúde da Pessoa Idosa (11), eram encaminhadas ao ambulatório de referência em geriatria e gerontologia situado na Região Administrativa de Taguatinga, por meio do sistema de regulação de consultas (SISreg).

No ambulatório, realizou-se consulta interdisciplinar com médico geriatra, enfermeiro, fisioterapeuta e nutricionista devidamente capacitados para essa finalidade. Os profissionais tomavam nota sobre os medicamentos em uso, aspectos socioeconômicos, comorbidades clínicas previamente diagnosticadas, além de queixas específicas relacionadas à vulnerabilidade descritas na Caderneta de Saúde da Pessoa Idosa do Ministério da Saúde, como o número de quedas no último ano, a prática de atividade física regular (aqui foram considerados ao menos 150 minutos de prática semanal de qualquer modalidade), o índice de massa corporal (IMC), a existência de incontinência urinária, a dependência para as atividades de vida diária e a identificação do fenótipo sarcopenia.

As consultas foram realizadas contando com a participação dos familiares e cuidadores das pessoas idosas. Inicialmente, realizou-se entrevista para levantamento de perfil com questões relacionadas à renda, ao estado civil e à escolaridade. Houve também a ratificação de informações contidas no motivo do encaminhamento, como presença de incontinência urinária, uma ou mais quedas nos últimos seis meses antes da anamnese e número de medicamentos em uso.

### Critérios de inclusão e exclusão

Como critério de inclusão, adotaram-se as pessoas idosas que estavam em condições de vulnerabilidade segundo critérios da Caderneta de Saúde da Pessoa Idosa do Ministério da Saúde, identificados pelo médico de família e equipe de estratégia de saúde da família durante as ações do matriciamento (11). Como critérios de exclusão, adotaram-se as pessoas idosas que apresentassem sequelas de doenças neurológicas (doença cerebrovascular, parkinsonismos, demência, entre outras), além de sujeitos com amputações, uma vez que tais agravos estão relacionados à sarcopenia secundária ao desuso atribuído a tais condições.

### Variáveis

Para o diagnóstico de sarcopenia, utilizou-se a recomendação proposta pelo consenso europeu por meio de algoritmo que leva em consideração três critérios distintos: a força muscular, a quantidade ou qualidade da massa muscular e o desempenho físico (3).

O teste de força de preensão palmar, feito por meio de dinamômetro hidráulico da marca Jamar, foi utilizado como marcador da força muscular global na pessoa idosa. O teste foi realizado com o sujeito sentado em uma cadeira sem apoiar as costas nesta, enquanto mantinha o cotovelo flexionado. Na mão dominante, foram feiras três medidas com intervalo de um minuto entre elas. Foi considerado sarcopênico caso as medidas apresentassem valores inferiores a 27 kg/F para homens e 16 kg/F para as mulheres (12,13).

Para avaliar a qualidade da massa muscular, mensurou-se a circunferência da panturrilha com o idoso sentado em uma cadeira, pernas relaxadas e pés apoiados no chão, joelhos fletidos a 90º. Após identificar a região mais protuberante das pernas, por meio de fita métrica inelástica, realizou-se a perimetria, sendo considerada como risco aqueles indivíduos que apresentassem valores iguais ou inferiores a 33 cm para mulheres e 34 cm para homens (14).

O desempenho físico, medido pelo teste de velocidade habitual de marcha, foi realizado em um corredor, onde o idoso foi orientado a deambular em velocidade habitual, podendo utilizar dispositivo auxiliar à locomoção. Mensurou-se o tempo transcorrido para deslocar-se por três metros, sendo desconsiderados os tempos de aceleração e de desaceleração, indicando risco as velocidades menores que 0,8 m/s (15). 

Para classificar se os idosos eram sarcopênicos, utilizaram-se os critérios preconizados pelo consenso europeu. Neste, os sujeitos que apresentassem redução de força muscular e qualidade da massa muscular foram classificados como sarcopênicos. Para serem definidos como sarcopênicos graves, o idoso deveria apresentar também redução do desempenho físico (3).

As variáveis antropométricas foram mensuradas em altura (cm) e peso (kg) por meio de balança com estadiômetro da marca Filizola, para em seguida calcular o IMC. Utilizou-se a recomendação de Lipschitz, classificando idosos com valores menores que 22 kg/cm^2^ como baixo peso e valores entre 22 e 27 kg/cm^2^ como sujeitos eutróficos. O idoso com IMC maior que 27 kg/cm^2^ foi classificado com excesso de peso, sendo estas medidas mais sensíveis para a saúde pública (16).

Em seguida, aplicou-se o índice de Barthel para a identificação do grau de independência funcional, no qual se utilizou a versão validada e adaptada transculturalmente à população brasileira, cujo ponto de corte foi maior ou igual a 60 para determinar os sujeitos como independentes para as atividades de vida diária. Para identificar a presença de sintomas referentes à incontinência urinária, utilizou-se o ritmo urinário do índice de Barthel, o qual considerou continente o idoso que alcançasse 10 pontos e incontinente aquele que alcançasse 5 ou 0 pontos (17). 

Aplicou-se questionário com questões relacionadas a aspectos socioeconômicos (entrevista para o levantamento de perfil com questões sobre renda, estado civil, escolaridade), presença de incontinência urinária, uma ou mais quedas nos últimos seis meses antes da anamnese, número de medicamentos em uso e nível de atividade física.

### Métodos estatísticos

Para análise estatística, utilizou-se o software Stata versão 15. Na análise descritiva, as variáveis foram ponderadas segundo média, desvio-padrão, além de proporções. Foram estimados a razão de prevalência para sarcopenia e o intervalo de confiança 95%. 

Utilizou-se a sarcopenia como variável independente e, por meio dela, usou-se o teste de Poisson para estabelecer os fatores associados por meio da razão de prevalência e do intervalo de confiança 95%.

## Resultados

Durante o período de coleta, foram acolhidas 900 pessoas idosas encaminhadas pelas unidades básicas de saúde à policlínica. Destas, 240 foram excluídas por possuir sequela de acidente vascular cerebral, 50 tinham diagnóstico confirmado de doença de Parkinson, 80 apresentavam déficit cognitivo grave (síndrome demencial) e 30 haviam sofrido alguma amputação de extremidade. Quinhentos indivíduos foram avaliados e classificados conforme o fenótipo da sarcopenia (Figura 1).

**Figura 1:** Diagnóstico e prevalência de sarcopenia de acordo com o algoritmo da European Workin Group of Sarcopenia in Older People

Houve prevalência e fatores associados à sarcopenia em homens e mulheres encaminhados pela Atenção Primária, sob a ótica de questões socioeconômicas e comportamentais, o que evidenciou fatores de risco, como faixa etária >70 anos, e fatores de proteção, a exemplo de ter um companheiro e ter o IMC eutrófico ou excesso de peso (Tabela 1). A prática de exercícios físicos confirmou-se como fator de proteção para a sarcopenia com relação às mulheres.

**Tabela 1:** Razão de prevalência (RP) e intervalo de confiança de 95% (IC95%) bruta e ajustada da sarcopenia em homens e mulheres encaminhados pela Atenção Primária sob a ótica de questões socioeconômicas e comportamentais. Brasília, 2017 (n=500)

	Mulheres (n=128)		Homens (n=33)	
	RP (IC95%)	p-valor	RP (IC95%)	p-valor
Aspectos socioeconômicos				
**Faixa etária** (anos)				
60-69	1,00		1,00	
70-79	1,77 (1,29; 2,41)	<0,001	3,37 (1,13; 10,17)	<0,051
>80	2,55 (1,91; 3,40)	<0,001	9,23 (3,35; 25,38)	<0,001
**Anos de estudo**				
Sem estudo	1,00		1,00	
≤8	0,83 (0,63; 1,08)	0,18	0,93 (0,32; 2,69)	0,891
>8	0,95 (0,69; 1,29)	0,75	0,25 (0,60; 1,08)	0,060
**Vivem com companheiro**				
Sim	0,77 (0,60; 0,99)	<0,05	0,39 (0,19; 0,82)	<0,050
Não	1,00		1,00	
**Renda familiar**#				
1-2 salário	1,00		1,00	0,332
>3 salários	1,01 (0,79; 1,31)	0,87	0,67 (0,30; 1,48)	
Variáveis comportamentais				
**Atividade física**				
Sim	0,45 (0,31; 0,66)	<0,001	1,57 (0,91; 2,72)	0,100
**Índice de Massa Corporal** (IMC) (kg/cm^2^)				
Baixo peso	1,00		1,00	
Eutrófico	0,51 (0,40; 0,64)	<0,001	0,46 (0,29; 0,73)	<0,001
Excesso de peso	0,25 (0,19; 0,34)	<0,001	0,18 (0,07; 0,43)	<0,001

A incontinência urinária apresentou-se como fator de risco para a sarcopenia em ambos os sexos com relação aos aspectos clínicos (Tabela 2). Para as mulheres, os principais fatores de risco para o desenvolvimento da sarcopenia foram tomar mais que cinco medicamentos e ter cardiopatia e osteoporose. Curiosamente, aquelas que tinham problemas articulares ou hipotiroidismo não tiveram sarcopenia. Para os homens, os fatores que identificavam a presença do fenótipo foram problemas visuais e ocorrência de quedas nos últimos seis meses antes da anamnese.

**Tabela 2:** Razão de prevalência (RP) e intervalo de confiança de 95% (IC95%) bruta e ajustada da sarcopenia em homens e mulheres encaminhados pela Atenção Primária quanto a aspectos clínicos relacionados à sarcopenia. Brasília, 2017 (n=161)

	Mulheres (n=128)		Homens (n=33)	
	RP (IC95%)	p-valor	RP (IC95%)	p-valor
**Medicamentos em uso**				
≤4 medicamentos	1,00			
>5 medicamentos	1,32 (1,07; 1,62)	<0,001	1,12 (0,72; 1,73)	0,611
**Comorbidades**				
Hipertensão arterial	1,26 (0,97; 1,65)	0,077	0,78 (0,48; 1,25)	0,311
Diabetes	1,08 (0,87; 1,34)	0,4799	0,82 (0,51; 1,33)	0,435
Cardiopatia	1,27 (1,08; 1,61)	<0,050	0,73 (0,42; 1,28)	0,289
Osteoporose	1,42 (1,16; 1,75)	<0,001	1,10 (0,53; 2,29)	0,789
Hipotiroidismo	0,45 (0,31; 0,66)	<0,001	1,57 (0,91; 2,72)	0,100
Depressão	0,94 (0,76; 1,17)	0,621	1,35 (0,84; 2,16)	0,201
Problemas articulares	0,67 (0,51; 0,87)	<0,050	1,12 (0,67; 1,88)	0,641
Câncer	1,54 (0,84; 2,85)	0,151	0,98 (0,49; 1,97)	0,966
Problemas visuais	1,96 (0,97; 3,96)	0,062	2,65 (1,32; 5,29)	<0,050
Problemas respiratórios	1,21 (0,78; 1,88)	0,389	0,26 (0,03; 1,90)	0,188
Queda nos últimos 6 meses?	1,12 (0,91; 1,38)	0,275	3,31 (1,98; 5,53)	<0,001
Tem incontinência urinária?	1,29 (1,04; 1,59)	<0,050	2,21 (1,42; 3,42)	<0,001

## Discussão

O estudo observou que, em determinada região do Distrito Federal, 163 pessoas idosas (32,6%) eram pré-sarcopênicos, 161 (32,2%) sarcopênicas e 38 (7,6%) sarcopênicas severas. Identificaram-se fatores de risco, como >70 anos e incontinência urinária, e fator de proteção, como o IMC elevado. Cardiopatias e osteoporose foram indicadas para as mulheres. Para os homens, déficit visual e ocorrência de quedas nos últimos seis meses foram apontados como fatores de risco.

Um estudo europeu utilizou um modelo de projeção com base na prevalência atual de sarcopenia e dados demográficos disponíveis para a população de 28 países da União Europeia. Sugeriu-se que o número de pacientes com sarcopenia aumentará de forma significativa nos próximos 30 anos, o que causará consequências relevantes, tornando-se um problema de saúde pública (18).

No oeste da China, foi comparada a prevalência de sarcopenia entre áreas urbanas e rurais. Entre 612 indivíduos avaliados como um todo, 9,8% apresentaram sarcopenia. Quando foi realizada a análise separada, a zona rural teve prevalência de 13,1%, quase o dobro da população residente em áreas urbanas (7,0%). Destacou-se que 61,0% dos idosos sarcopênicos residiam na zona rural (19).

A presente região no Distrito Federal é composta por chácaras, onde o acesso a serviços de saúde a nível secundário (policlínicas) para o acompanhamento longitudinal pode ser comprometido. A identificação do fenótipo da sarcopenia poderia ser implementando nas rotinas e atividades realizadas na Atenção Primária para a população idosa.

Na Austrália, em uma amostra com 162 idosos, observou-se que 25 (16,0%) destes foram classificados como sarcopênicos. O comportamento sedentário foi apontado como o principal elemento associado à diminuição da massa muscular, aumentando o risco de desenvolver a sarcopenia (20).

Neste estudo, as mulheres que faziam exercícios físicos não apresentaram sarcopenia, o que caracterizou o acesso à saúde como comportamento predominantemente feminino. São necessárias estratégias *in loco* para que os homens desde cedo – não apenas quando se tornarem idosos – busquem a Atenção Primária para cuidar da saúde, e não para tratarem apenas de doenças.

No que diz respeito ao estilo de vida, a prevenção do comportamento sedentário leva ao menor risco de desenvolvimento de morbidades em pessoas idosas decorrentes da sarcopenia, pois a realização de exercícios físicos por pelo menos 150 minutos semanais é fundamental para o desenvolvimento de massa muscular e força muscular. Observou-se que a dieta pobre em proteínas, além do tabagismo e do etilismo, leva à perda de massa muscular (21).

Verificou-se que pessoas idosas que possuíam maior renda praticavam regularmente alguma atividade física, visto que os sujeitos de baixa renda consideravam a atividade ocupacional como atividade física (22-24). Os sujeitos com baixa renda tendiam a adotar outros hábitos menos saudáveis por falta de informações, como o tabagismo e o etilismo, o que contribuiu para o desenvolvimento de comorbidades ou doenças crônicas independentemente do sexo. Esses fatores podem contribuir para o aumento das taxas de prevalência de sarcopenia para os próximos anos (22-24).

Apesar de os resultados deste estudo demonstrarem maior prevalência de sarcopenia quando comparado a outras populações, vale lembrar que pessoas idosas muitas vezes apresentam vulnerabilidade quer seja no acesso aos serviços especializados, quer seja por questões culturais sobre o processo de envelhecimento, como se perder força e urina fosse normal para a idade. Há a necessidade de que, na Atenção Primária à Saúde, que está mais próxima dessa população, se desenvolvam estratégias identificando o risco da sarcopenia e o seu acompanhamento por meio de ações preventivas, educativas e acompanhamento.

A sarcopenia influencia negativamente a recuperação da independência na micção e defecação, independentemente do nível físico e cognitivo (25,26). A detecção precoce da sarcopenia e o tratamento através de modificações na dieta e na redução do comportamento sedentário devem ser implementados para prever e maximizar a melhoria na independência, visto que o controle de esfíncteres é atividade de vida diária (25,26).

Não há consenso na literatura sobre determinado sexo estar mais predisposto à sarcopenia que outro. Nos homens, o declínio dos esteroides sexuais é muito mais lento do que nas mulheres, o que pode ser fator importante para explicar a prevalência significativamente maior de sarcopenia nas mulheres entre 60 e 70 anos de idade. Nos homens, após a oitava década de vida, as concentrações de testosterona diminuem rapidamente, o que pode contribuir para o surgimento da sarcopenia. As mulheres, em média, têm a expectativa de vida mais longa do que os homens devido à cultura de cuidado e prevenção, buscando regularmente os serviços de saúde como a Atenção Primária (27,28).

O risco maior de se tornar sarcopênico é quando a pessoa idosa apresenta mais de dois agravos (multimorbidades), sobretudo diabetes, o que pode causar aumento da resistência insulínica e, com isso, intensificar a perda da massa muscular (7). 

Não há consenso sobre a relação de causa e efeito entre sarcopenia e osteoporose. Para o desenvolvimento de matriz óssea, é necessária a contração muscular e a implementação de exercícios com carga muscular, que também são indicados para o tratamento da sarcopenia. Entre as estratégias relacionadas à prevenção de quedas, que é fundamental para a prevenção de fraturas por conta da osteoporose, exercícios de força também são indicados (29,30). 

Encontrou-se a correlação o risco de quedas e a sarcopenia em 27,0% dos homens e 20,0% das mulheres (31). Um fato que pode ter apontado o risco de queda maior nos homens quando comparado às mulheres foi a prática regular de exercícios físicos que, no caso delas, é frequente e manifestou-se como fator de proteção tanto para a sarcopenia como para as dores articulares.

O quesito pessoas idosas que têm um companheiro foi apontado no estudo como fator de proteção para sarcopenia, demonstrando a necessidade da convivência social como necessidade humana básica. Ter a companhia de alguém pode atuar como estratégia para a motivação e facilitar o convencimento quanto à prevenção de comportamentos de risco, principalmente por conta das orientações de saúde e cuidados que são parte da rotina dos atendimentos na Atenção Primária. Existem também as práticas de convivência, como o Hiperdia, e as práticas integrativas e complementares em saúde que podem contribuir para a prevenção da sarcopenia, uma vez que a pessoa idosa precisa ir à unidade básica de saúde para participar e conviver; logo, o risco de isolamento social também diminui.

Ainda não há diretrizes clínicas brasileiras para o tratamento da sarcopenia, contudo a prevenção por meio de exercícios físicos regulares e alimentação equilibrada proporcionam a qualidade de vida para um envelhecimento bem-sucedido. A sarcopenia acarreta enormes custos para a sociedade; por sua vez, o aumento da expectativa de vida e o consequente aumento da população idosa causaram prevalência importante de sarcopenia. A gestão custo-efetiva dessa doença tornou-se particularmente importante (32). 

A Portaria nº 635/2023 do Ministério da Saúde estabeleceu o incentivo financeiro federal de implantação e custeio para as equipes multiprofissionais na Atenção Primária. Esse incentivo surgiu no momento de reconstrução focado na atenção básica da pessoa ao longo do curso de vida, por meio do fortalecimento das ações interprofissionais e incorporação de tecnologias e inovações em saúde para promover o cuidado integral da população. Isso contribui para ampliar o escopo de práticas e a resolubilidade na própria Atenção Primária (33).

Como limitações deste estudo, informações sobre estilo de vida como tabagismo, etilismo e lazer não foram levantadas, bem como raça/cor da pele, religião e estado nutricional. Essas informações poderiam determinar outros fatores que podem influenciar no desenvolvimento da sarcopenia. Também não foi levantado quais foram as unidades básicas de saúde responsáveis pelo encaminhamento das pessoas idosas com sarcopenia, fato que poderia auxiliar na identificação das áreas de maior vulnerabilidade para estratégias de matriciamento.

Este estudo observou a prevalência de 32,2% de sarcopenia e 7,6% de sarcopenia grave em idosos atendidos pela Atenção Primária em determinada região do Distrito Federal. Caracterizou-se real problema de saúde pública na atualidade e identificaram-se como fatores de risco ter >70 anos e incontinência urinária e como fator de proteção ter o IMC elevado.

Cardiopatia e osteoporose foram fatores de risco para sarcopenia em mulheres e, para os homens, foram problemas visuais e ocorrência de queda. Como fatores de proteção para sarcopenia para as mulheres, consideraram-se ter um companheiro e realizar atividade física regular.

## Data Availability

O banco de dados poderá ser disponibilizado em planilha de Excel mediante a solicitação ao autor correspondente.
